# Diversity in energy transition: a multi-case study on DIY solar energy in Germany

**DOI:** 10.3389/fsoc.2026.1821513

**Published:** 2026-07-15

**Authors:** Samantha Schwickert, Melanie Jaeger-Erben, Astrid Preis, Jenja Tscherkaschin

**Affiliations:** Institute for Philosophy and Social Sciences, Sociology of Technology and the Environment, Brandenburg University of Technology Cottbus–Senftenberg, Cottbus, Germany

**Keywords:** citizen energy, energy transition, feminist energy, gender and energy, inclusion, just energy transition, masculinities and energy

## Abstract

The transition to renewable energy systems is not only a technological challenge but also a deeply social and cultural process with significant implications for intersectional equality. Citizen-led energy initiatives are often portrayed as ideal spaces for participatory engagement, yet they can reproduce exclusionary dynamics, particularly regarding gender-related injustices. With a particular focus on FLINTA*-persons this study examines how citizen-led, Do-It-Yourself (DIY) photovoltaic (PV) initiatives in Germany translate normative commitments to diversity and justice into everyday organizational practices. Using a comparative qualitative case study approach, six initiatives were analysed through document-analysis, interviews, and participant observations to explore reflexive orientations—how actors understand gender, diversity, and justice—and performative practices—how these orientations are enacted through strategies, activities, and organizational forms. Findings reveal persistent gaps between diversity aspirations and implementation, shaped by limited resources, organizational constraints, and socio-cultural barriers. Patriarchal norms continue to limit FLINTA*’s participation, even in initiatives explicitly committed to inclusivity, while masculinities within these initiatives often reproduce subtle exclusionary practices. To capture these dynamics, we introduce the concept of ecological-hegemonic masculinity, describing configurations in which men, despite valuing renewable energy, social benefits, and care-oriented narratives, continue to align with hegemonic norms of autonomy, control, and guidance. Furthermore, the study contributes a novel perspective linking reflexive and performative approaches to inclusion and highlights conditions under which diversity efforts succeed or falter. From a practical standpoint, creating protected spaces for FLINTA* and other marginalized groups, alongside sustained financial and institutional support, is essential for moving inclusion from individual motivation to structurally embedded practice. These insights advance both theoretical understanding and actionable strategies for achieving socially just energy transitions.

## 1 Introduction

The transition toward low-emission and renewable energy systems is increasingly understood not only as technological change, but as a profound societal project with far-reaching implications for social fairness and legitimacy. It raises fundamental questions of intergenerational, intersectional, and global justice, particularly concerning the distribution of costs and benefits, access to decision making, and the recognition of diverse forms of knowledge (Bell et al., 2020; Bidwell and Sovacool, 2023; Laborgne and Radtke, 2023). Thus, achieving a just energy transition cannot be reduced to technological innovation and infrastructural change. Rather it depends on social, cultural, and institutional processes that shape who participates, whose knowledge is recognised and legitimate, and which norms are reproduced or contested within energy systems. Within this broader context, citizen engagement has emerged as a key governance strategy for aligning decarbonisation with democratic goals. Citizen or grassroots energy initiatives, in particular, are often portrayed as an ideal type of participatory engagement. Yet empirical research indicates that, despite these aspirations, such initiatives can reproduce exclusionary logics in their governance and participation practices, particularly in terms of gender diversity (Dudka, 2024; Fraune, 2018; Karakislak et al., 2023; Łapniewska, 2019; Tsagkari, 2022).

Building on feminist and intersectional theories in energy transformation (Bell et al., 2020; Sovacool et al., 2023; Walk, 2024), this paper aims to identify the particular practices and social mechanism through which grassroots renewable energy initiatives reproduce but also challenge social inequalities, particularly gendered and intersectional power relations. While existing research had made an important contribution to conceptualising justice and inclusion in energy transitions, there remains little understanding of how such normative commitments are translated into everyday organisational practices in grassroots initiatives and how they are enacted and negotiated in their particular dynamic socio-technical settings. Instead of focusing on cis women alone, we use the broader term FLINTA* (“female, lesbian, intersex, non-binary, trans and agender people”) to differentiate better between structurally disadvantaged groups in a patriarchal gender order. While many “women in energy” approaches implicitly focus on cis, heterosexual women, we aim to understand better how inter, non-binary, trans and agender people, as well as lesbians, face specific forms of discrimination (for example transphobia, cis-sexism, homophobia) on top of sexism. We assume that in sectors like energy cooperatives, where gender equality work has traditionally concentrated on “women vs. men,” nderrepresentation and exclusion affect a wider spectrum of marginalized genders, and that strategies for a gender-just, democratic energy transition need to address all of them. Using FLINTA* therefore signals that the focus is on dismantling patriarchal power structures for all marginalized genders, rather than improving the position of (some) women while leaving other gendered exclusions intact. We further have deliberately chosen to adopt a regional perspective here and focus on Germany, partly because we believe this allows us to better highlight regional and national specificities, and partly because Germany is often cited as a good example of the energy transition that is worth examining critically. Based on this, the paper aims do develop empirically grounded strategies for inclusive participation and equitable governance in citizen-led energy transitions, particularly focusing the “meso-level” of how inclusion and equity is routinised and institutionalised (or not) in organisations.

To this end, we examine and comparatively evaluate six different renewable energy initiatives in Germany using a qualitative case study approach that combines problem-focused interviews with participant observation. The analysis addresses the following research questions: How do key persons in grassroot citizen energy initiatives (CEI) relate to or reflect on gender, diversity, and justice? How does reflexivity translate into their activities/performances? What performative challenges and solution-oriented practices emerge with regard to the participation of FLINTA* persons, and how do these interact with everyday organisational practices and socio-technical settings?

In the following, we will first provide an overview of the theoretical background (Chapter 2), before presenting our methodological design (Chapter 3). Building on this, we will present our empirical comparative case analysis, focusing on reflexive (Chapter 4.1) and performative dimensions of inclusion (Chapter 4.2). Next, we will develop empirically grounded patterns of reflexive-performative inclusion and transformative depths (Chapter 4.3). Finally, we discuss three interrelated factors that limit and challenge diversity endeavours in processes of socio-technical transition.

Our contribution extends existing debates on diversity in energy transitions by linking *reflexive* orientations (how initiatives understand gender, diversity, and justice) with *performative* dimensions (how these understandings are enacted through strategies, practices, and organizational forms) and sheds light on the persistence of limited diversity energy transitions.

## 2 Theoretical background

The transition toward renewable energy systems constitutes a complex socio-technical transformation that extends well beyond the reconfiguration of infrastructures and institutions. It reshapes how energy is produced, distributed, accessed, and used, while simultaneously influencing social relations and power structures (Barry et al., 2015; Hoicka, 2023). These dynamics are commonly examined through energy justice frameworks, which pose normative questions about how distribution, recognition and participation are linked in energy systems (Siciliano et al., 2021; Sovacool et al., 2023) and which offer a key evaluative lens for our analysis.

At the same time, a justice-oriented transformation of socio-technical systems requires attention to how unjust power relations and social inequalities are embedded in, and reproduced through, these systems (Siciliano et al., 2021). To address these gendered, embodied and intersectional dimensions and to shift the analytical focus from abstract justice principles to the relational and performative reproduction of injustices, this paper draws on intersectional feminist and masculinity theories. By foregrounding diversity, equity and inclusion as dynamic and contested practices (Sharma et al., 2025), we seek a more fine-grained analysis of power relations within energy transition.

In the following, we first specify our justice framework (2.1) and situate it within the German energy transition and field of citizen energy initiatives (2.2). We then elaborate intersectional feminist and masculinity-informed perspectives (2.3), before introducing our analytical distinction between reflexive and performative inclusion in self-organised initiatives (2.4).

### 2.1 Just energy transition

Building on energy justice scholarship, we focus on four dimensions of justice that structure our normative framework for analysing energy transitions. Distributional justice centres on the fair distribution of energy resources and the associated benefits and costs (Büchs et al., 2023), asking who gains access to affordable, clean and reliable energy and who bears environmental burdens. Procedural justice concerns transparent, inclusive and participatory decision-making, in which affected communities and marginalized groups are actively involved in the planning, implementation and governance of energy projects (Jenkins et al., 2016; Sovacool et al., 2023). Recognitional justice highlights the importance of acknowledging the diversity of perspectives, identities and needs of different social groups and of reducing discrimination and misrecognition by integrating these perspectives into energy-related decision-making (Van Uffelen, 2022). Finally, compensatory justice addresses reparations for past damage and compensation for communities that are disproportionately affected by environmental destruction and economic restructuring and is therefore particularly relevant at global and transnational scale (Feenstra and Özerol, 2021).

By design, CEIs are expected to address at least distributional, procedural, and recognitional justice as it is grounded in a decentralized, renewable, and community-based approach (Blome-Drees et al., 2024; Shejale et al., 2025). Despite its emancipatory orientation, CE practices tend to remain socially exclusive, with membership and leadership predominantly drawn from more educated and affluent milieus—often older, white men (Goedkoop et al., 2025; Lazoroska et al., 2021). This social selectivity limits CE’s ability to fully realize the three justice dimensions of distribution, participation and recognition, while compensatory justice is largely absent from CE discourses and practices. Discriminatory mechanisms and the reproduction of inequality are connected, we therefore focus on both dynamics within initiatives that are formally committed to justice-oriented goals.

### 2.2 Diversity in the German energy transition

Germany represents a mature energy-transition context with well-established policy frameworks and a long-standing bottom-up energy transition movement. This makes it a particularly relevant setting for examining how inequalities between genders and different social groups persist, even in a country widely regarded as progressive in its energy, equity, and sustainability ambitions.

Recent data show that patterns of gender inequality remain pronounced across key arenas of the German energy transition (PwC, 2025). While the share of women has increased in some political functions and research institutes since 2021, it has declined in public utilities, renewable energy companies and advocacy groups. In the conventional energy sector, women continue to be underrepresented both in vocational training and in leadership positions (Von Kemfert and Egerer, 2017; Luh and Löw Beer, 2021). Political offices, relevant for the energy transition, such as the Parliamentary Committee on Economic Affairs and Energy, the Federal Ministry for Economic Affairs and Climate Action (BMWK),^1^ and key industry associations, are likewise dominated by men (Groneweg and Habersbrunner, 2024). In the renewable energy sector, the proportion of women in top management roles decreased since 2021 from 15.5 to 14.3%, revealing significant gender disparities (PwC, 2025).

Within this context, CE is often associated with the potential to redistribute power and decision-making in the energy system. However, according to current research, CE in Germany is not inherently more just than the conventional energy sector (Karakislak et al., 2023; Tsagkari, 2022; WECF, 2020). Limited participation and representation of women permeate nearly all areas of CE: from roles as prosumers (e.g., balcony PV systems, tenant electricity models) and involvement in local energy cooperatives—which often rely heavily on voluntary labour—to more professionalized domains such as project development, entrepreneurship, and the management of CE enterprises (Karakislak et al., 2023; Radtke and Ohlhorst, 2021; Yildiz, 2014). Similar patterns have been identified for other organizational forms of CE, such as limited liability companies (GmbHs) or civil law partnerships (GbRs), where women tend to hold fewer memberships and demonstrate lower levels of financial participation (Fraune, 2015). Inequalities in CE are shaped in an intersectional manner, as not only women, but also individuals with lower levels of formal education and low-income households are consistently underrepresented (Bögel et al., 2023). Bögel et al. (2023) further demonstrate that the creation of a shared group identity can empower marginalised groups to engage in energy transition projects, while low-threshold participation formats such as balcony photovoltaic modules can enable tenants to become involved.

But despite growing academic interest in just and inclusive (energy) transitions, only a limited number of practical initiatives and projects explicitly aim to increase inclusion within the energy transition (ibid.). Moreover, empirical research still often relies on a binary understanding of gender and neglects a broader gender spectrum (Cannon and Chu, 2021; Fathallah and Pyakurel, 2020; Søraa et al., 2020). Binary notions are frequently combined with stereotypical roles, attributes or preferences ascribed to “men” and “women,” even where these are acknowledged as socially constructed (Ahlborg et al., 2024; Fathallah and Pyakurel, 2020). Evidence from other countries suggests that, i.e., indigenous, Black and Latin individuals are underrepresented in CE relative to their share of overall population (Hoicka et al., 2021) and that intersecting forms of racism, sexism, and classism have exclusionary effects on participation (Hoicka, 2023; Sharma et al., 2025).

### 2.3 Intersectional feminism and masculinity studies in energy transition research

Intersectional feminist perspectives on commons, technology, and sustainability demonstrate that socio-technical transformations are deeply embedded in power relations, norms, and inequalities, and thus never gender-neutral (George et al., 2018; Haraway, 1996; Schultz et al., 2015; Wolfram and Kienesberger, 2023). In this study, we conceptualize intersectional social research as a multidimensional analysis of power relations that attends to the interlocking effects of gender, sexuality, race, class, ability, and bodily norms within capitalist societies (Ganz and Hausotter, 2020; Winker and Degele, 2009). Such an approach enables a more nuanced understanding of individual experiences, social relations, and processes of exclusion and discrimination, while also highlighting possibilities for agency, emancipation, and collective capacity to act (Winker and Degele, 2009).

Within this broader intersectional perspective, we draw on liberal, ecofeminist, postmodern, and postcolonial theories as key narratives of a gender-just transition (Walk, 2024), which conceptualize gender inequality as unequal access to opportunities and resources, link the oppression of women to the exploitation of nature, destabilize binary and essentialist understandings of gender and situate gendered injustices within global histories of colonialism, racism and capitalist extraction (i.e., Crenshaw, 1991; Kanning et al., 2016; Lenz, 2019; Lorber, 2010; Mies and Shiva, 2016; Mishra, 2013). Additionally, previous studies have shown that behavioural changes, especially ones regarding basic life resources, are oftentimes feminized, while ecological technologies are dominated by men. Drawing on the concept of hegemonic masculity, which describes practices that enable and sustain the structural dominance of men over women (Connell and Messerschmidt, 2005), a newer strand of feminist scholarship examine gendered power formations that resist socio-ecological transformation. These dominant configurations of masculinity—conceptualised as *petromasculinity* (Daggett, 2018) or *industrial/breadwinner masculinities* (Hultman and Pulé, 2018)—are historically and symbolically intertwined with fossil fuel–based modernity and authoritarian political imaginaries.

Contemporary energy transitions toward renewable energy unfold within a tension between ecological modernisation and more far reaching social-ecological transformation (Bell et al., 2020). Eco-modern perspectives frame sustainability primarily as a technological and management problems to be solved through innovation, policy optimisation, and market-based solutions, while reproducing masculinist forms of authority, expertise and control (Hultman and Pulé, 2018). In contrast, Hultman’s (2021) notion of ecological masculinities, drawing on masculinity politics, deep ecology, ecofeminism, and ethics of care, imagines more relational, justice-oriented, and transformative forms of climate and energy engagement. Masculinities are enacted in diverse ways by men, women and non-binary people and intersect with age, generation, class and other social hierarchies that structure power and privilege (Scharnigg and Martin, 2024). Although citizen energy initiatives may provide fertile ground for the enactment of ecological masculinities this assumption has not been systematically examined and is crucial for understanding gendered dynamics of participation and power in just energy transitions.

Taken together, these intersectional feminist and masculinity-related research perspectives trace how overlapping forms of discrimination (e.g., sexism, racism, classism, ableism, heteronormativity, postcolonial inequalities) shape whose labour in energy governance is rendered visible and valued and whose care and coordination work remains backgrounded, and they call for expanding energy-transition imaginaries beyond technocratic rationalities toward relational, care-oriented and plural understandings of sustainability (Crenshaw, 1989; Hooks, 2012; Martinez-Reyes et al., 2025; Sovacool et al., 2023).

### 2.4 How the reflection and performance of inclusion enable transformation: a practice-based analytical framework

Building on the perspectives outlined above, we understand diversity and inclusion as going beyond mere numerical representation of marginalised groups (Krell and Sieben, 2011; Sharma et al., 2025). Whereas representation and integration focus on who is present in communities, boards or organisations, diversity and inclusion additionally concern fairness, equal opportunities, and the substantive integration of different groups’ knowledges and perspectives into decision-making and practice (WECF, 2020).

Conceptually, our perspective implies several methodological premises:

First, that citizen energy initiatives must be analysed through their practices, understood as the co-constitution of meanings and actions rather than as isolated attitudes or outcomes. Building on Giddens’ understanding of social life as recursively produced through knowledgeable, reflexive action (Giddens, 1984), we conceptualise practices in citizen energy initiatives as composed of both reflexive and performative dimensions of habitus and bodily practices. We operationalise the discursive consciousness as reflexivity that shows in the ways in which actors monitor, interpret and (re)articulate their own conduct and its conditions, for example how they frame problems of gender, diversity and justice, formulate goals and diagnose exclusion in light of broader structural contexts. Performative aspects, by contrast, concern the concrete enactment of these understandings in situated “doings”: the routines, organisational arrangements and material interventions through which inclusion is pursued in everyday practice. This reflexive/performative distinction allows us to analyse how citizen energy initiatives not only talk about inclusion and justice, but also how they reproduce or transform existing socio-technical structures through their practical activities (see our preliminary work on the framework in Schwickert et al., 2025).

Second, we assume that these practices are embedded in intersectional power relations and that the question of transformation concerns not merely the presence of ‘inclusive’ measures but their depth in relation to existing socio-technical structures and how these structures are challenged and changed (Winker and Degele, 2009). In order to conceptualize different degrees of change more precisely, we draw on Dolata’s theory of socio-technical transformation (Dolata, 2011). Dolata distinguishes between incremental modifications within existing socio-technical arrangements and more far-reaching, radical transformations that realign the institutional, organizational, and normative foundations of a field. Building on this distinction, we use the notion of ‘transformative depth’ to analyse whether and how citizen energy initiatives move beyond merely incremental change that has little impact on the overall structure—such as diversity-sensitive language, toward architectural transformation that aims to reorganize roles, rules, decision-making structures, and the allocation of resources—such as mandatory quotas, new committees, or structurally embedded safe-space formats and therefore challenges structural inequalities in energy governance. Inclusion is thus conceptualized as a processual, context-sensitive, and performative practice a continuous negotiation embedded in social relations and institutional settings rather than a fixed status (Ahmed, 2017). Table 1 shows how both analytical lenses were applied.

**Table 1:** Analytical lenses on inclusion practices in citizen energy initiatives.

Aspect	Reflexive (how initiatives think/talk)	Performative (what initiatives do)
Focus of analysis	Problem framings of gender/diversity/justice; goals, diagnoses of exclusion; self-understandings (e.g., FLINTA*, ecological masculinities)	Concrete strategies, routines, organisational forms, participation formats, role allocations
Key questions	How are inclusion and justice understood and justified? Whose experiences and inequalities are recognised or silenced?	How are inclusion and justice enacted in practice? Who participates how, with which resources and visibility?
Transformative depth: incremental	Reflexive shifts that remain within existing categories and institutional logics (e.g., adding diversity language, general equality goals)	Add-on measures that modify existing arrangements without changing underlying structures (e.g., one-off FLINTA* events, voluntary quotas)
Transformative depth: architectural	Reflexive re-articulation of mission and problem definitions that questions dominant norms and power relations (e.g., intersectional, feminist, decolonial reinterpretation of CE’s purpose)	Structural reorganisation of roles, rules and resource distributions (e.g., binding quotas in statutes, new decision bodies, redistributive participation models) that realign socio-technical and institutional foundations

## 3 Methodology

This study follows a qualitative multiple case study design to explore how different reflexive orientations toward gender, diversity, and justice manifest in the practices of CE initiatives.

### 3.1 Choice of cases and case description

The selected six case studies represent diverse models of Do-It-Yourself (DIY) solar and citizen-led renewable energy initiatives in Germany, differing in scale, organizational form, and social composition. Cases include associations, action research projects, and neighbourhood initiatives that aim to expand renewable energy production and citizen participation. The case selection followed a multi-stage sampling process. Together with the project’s practice partners, we compiled a database of citizen-led solar energy initiatives in Germany, drawing on practitioners’ field-based knowledge and complementary desk research. In a second step, initiatives were assessed in relation to the research questions and discussed iteratively within the research team. Particular attention was paid to initiatives’ focus on citizen participation in solar energy production, variation in organizational forms, and their engagement with questions of gender and participation. From this pool, six cases were purposively selected to capture variation across organizational settings while ensuring comparability within the German solar energy context. We only focus on Germany as a research context (see Chapter 2.2).

#### 3.1.1 Case A

The initiative has the overall goal to achieve a climate neutral and socially just city in Germany. Its approach is based on the concept of a donut economy and fulfils the Paris Agreement and the Sustainable Development Goals. The strategy is targeting neighbourhoods by supporting applications for balcony solar power plants and organizing neighbourhood groups around the topics of renewable energy, mobility, safety, health and community.

#### 3.1.2 Case B

The initiative has the overall goal to shape the shift toward renewable energy in Germany bottom-up by encouraging participation of individuals and neighbourhoods in climate change strategies. It is active in three fields: Installing balcony solar power plants in households in or around a city in Germany or offering consultations for such (1), installation of solar plants on buildings in teams consisting of experts and volunteers (2) and offering education around the topics of renewable energy and sustainability (3). The initiative has a special focus on the empowerment of FLINTA* people and therefore offers specific measures, e.g., FLINTA*-Workshops.

#### 3.1.3 Case C

The initiative was founded on the idea of combining ecological or environmentally oriented endeavours based on solidarity with practice. The aspired goal is to enhance the energy transition a city of Germany by focusing on three fields: Installing balcony solar power plants in households or offering consultations for such (1), installation of solar plants on buildings in teams consisting of experts and volunteers (2) and using ecological electricity only (3). The initiative has a specific focus on class and social status as a barrier and has therefore based their installation of balcony solar plants on a solidarity system, where customers who can afford it can pay a higher price to fund solar plants for people with less or little income.

#### 3.1.4 Case D

The initiative is based on two assumptions: the industry field of photovoltaics is systematically and with an increasing tendency lacking qualified personal (1) and people who are interested and educated on solar power have an influence on the people surrounding them (2). It therefore host educational camps across Germany to encourage access to work in this industry. The camps each consist of a theoretical part with lessons and exercises and a one-to-two-week training, where the trainees spend time on the building site. Each summer several camps take place in different cities across Germany, where many people successfully complete their education program and become photovoltaics installation assistants.

#### 3.1.5 Case E

The initiative has the overall goal to promote climate protection through distribution of solar power, which they aspire to reach through three fields of work: taking part in energy politics by, e.g., publishing statements (1), offering consultations for installation of solar panels (2) and organizing events to distribute information about solar panels in neighbourhoods (3).

#### 3.1.6 Case F

The research project focuses on the questions how a socio-ecological energy transition could look like, how a more sustainable production of energy, specifically through balcony solar power plants, impacts everyday- and community life and how it can be linked to other aspects of sustainability. During the project, individuals received balcony solar power plants for free to produce energy in their households, throughout this process they were supervised by scientistic personnel. The project centres networking and community: the goal is to establish a continuous communication between the participants and other interested people and to focus on underrepresented social groups, such as women.

### 3.2 Data gathering

Data were generated through desk research and document analysis, 13 semi-structured problem-centred interviews with initiative leaders and active members, five participant observations at events including ad-hoc interviews with participants, and an online feedback form, all conducted in 2025 (see Table 2). Documentary materials included websites, project descriptions, reports, and other publicly available materials produced by the initiatives. The interviewees hold positions as project coordinators, managers, founders, research assistants, board members and volunteers in the initiatives. Across all interviews conducted, we used a semi-structured interview guideline based on the problem-focused interview (Witzel and Reiter, 2012), that was adapted to the respective focus and stage of the project while maintaining conceptual continuity. All interviews were transcribed verbatim, and the participatory observations and ad-hoc interviews were documented through researcher memos. In addition, a short online feedback form was conducted to capture participants’ perspectives on inclusion and participation; six responses were collected, which provided useful complementary impressions of participant experiences. While interviews constituted an important source of data across the study, the empirical material was generated through the combination of interviews, participant observation, and documentary analysis. In Case D, no formal interview was conducted; however, data were generated through intensive participant observation during a multi-day event, supplemented by numerous informal conversations with participants and volunteers as well as documentary materials. These sources enabled the analysis of organisational practices and reflexive dimensions in a manner comparable to the other cases.

**Table 2:** Research method per case.

Case	Method	Amount
A	Interviews with employees and volunteers from the initiative	2
	Participatory Observation with ad-hoc Interviews with participants	1
B	Interviews with employees and volunteers from the initiative	3
	Participatory Observation with ad-hoc Interviews with participants	1
	Online feedback form	1
C	Interviews with employees and volunteers from the initiative	4
	Participatory Observation with ad-hoc Interviews with participants	1
D	Interviews with employees and volunteers from the initiative	0
	Participatory Observation with ad-hoc Interviews with participants	1 (for 4 days)
E	Interviews with employees and volunteers from the initiative	2
	Participatory Observation with ad-hoc Interviews with participants	1
F	Interviews with employees and volunteers from the initiative	2
	Participatory Observation with ad-hoc Interviews with participants	0
	Online feedback form	1

### 3.3 Data analysis

Data analysis followed a qualitative content analysis approach (Kuckartz, 2018), combining deductive categories derived from the reflexive–performative framework (see chapter 2.4) with inductive coding from the empirical material. Following the coding procedures proposed by Kuckartz (2018) we conducted several cycles of coding to develop and refine our category system. The initial deductive coding framework was developed collaboratively by the research team and subsequently applied independently by two researchers across the empirical material. Emerging inductive categories, coding decisions, and category definitions were discussed and refined through team meetings. Case interpretations were iteratively reviewed and revised within the author team to enhance analytical consistency. We applied this approach to our interview data, the materials generated through participatory observations, as well as to the initiatives’ websites and selected outreach or marketing materials. The use of multiple data sources, including interviews, participant observations, and documentary materials, enabled triangulation and contributed to the robustness of the findings. The analysis focused on how the initiatives conceptualize inclusion, which strategies they employ to promote diversity, and which barriers they face in implementation. Based on these findings, four patterns were developed following Kelle and Kluge (2010), capturing interpretive orientations (reflexive) and observable practices (performative) as interrelated dimensions of inclusive transformation. The development of the patterns followed a collaborative process in which case profiles and emerging types were discussed collectively and compared across cases.

## 4 Results

This chapter presents the results of the cross-case analysis, highlighting key similarities, recurring themes, and variations across the six investigated initiatives. The analysis focuses on how different organizational forms and social compositions shape practices of inclusion and exclusion in citizen-led and DIY solar projects. Drawing on our framework, we first examine how initiatives frame gender, diversity, and justice (reflexive dimension) and then how they enact these orientations in concrete practices and organisational arrangements (performative dimension), while recognizing that these dimensions inevitably overlap in both narrative and practice. On this basis, we assess the transformative depth of inclusion-oriented activities, distinguishing between more incremental (substitutive) and more architectural (structural) changes.

### 4.1 Reflexive dimensions of inclusion

Across all six initiatives, interviewees articulated a clear normative commitment to diversity and inclusion, frequently emphasising that “diversity could and should be higher than it is today in many, intersectional directions” and that the participation of women and FLINTA* persons is “important” and “highly valued.” At the same time, many representatives described inclusion as a demanding and resource-intensive task that competes with other priorities, and several admitted that they “do not yet know” how to design more inclusive formats or how to respond adequately to discriminatory behaviour. This general reflexive acknowledgement that inclusion matters, coupled with uncertainty about its concrete realisation, provides the backdrop for the following analysis of how initiatives frame participation, diversity and justice in more detail.

#### 4.1.1 Target group orientation and meanings of inclusion

Across all case studies, the question of who is being addressed—and how—plays a central role in shaping access to participatory formats in the energy transition. While some initiatives explicitly define their target groups, others work more implicitly, relying on language, visual communication, or personal networks to shape their outreach.

Initiatives differ considerably in how they conceptualize their audience. One initiative explicitly aimed to reach beyond the usual “pioneer users” and engage women and people with low incomes, deliberately advertising through local newspapers and using diversity-showcasing pictures and narratives to attract diverse people. Two other initiatives acknowledged that their core audience still consists of older, wealthier homeowners. However, both made efforts to appeal to younger or more diverse audiences through educational initiatives or idealistic more eco-conscious approaches, which are assumed to attract more women.

Three other initiatives explicitly aim to escape the stereotypical “eco-bubble.” One, for example, positions itself as inclusive of FLINTA* participants but also self-reflects that it potentially reproduces eco-scene aesthetics in their web design, which may limit broader outreach. As one interviewee put it:

*“When you look at our website, it’s all young white eco people. The men all have long hair, and the women have short hair. It says things like ‘collective’ and ‘non-profit association.’ I don’t know if people feel like they can relate to that.”* (I.2)

Another targets specific social groups such early adopters, tech-savvy youth and neighbourhoods. A third initiative aims at young people or people who want to change their subject interested in solar craftwork.

Nearly all interviewees framed gender predominantly in binary terms and refer mostly to women as an important target group. Two cases involve specific formats or events tailored to queer or trans audiences, such as a FLINTA* construction site or a FLINTA* solar camp. In these two instances, only one appeared to have the expertise to create spaces that go beyond tokenism; in the other, a TIAN* participant reported the impression that the format was designed primarily for cis women.

#### 4.1.2 Contextual framings: time, money, technology

Several sociocultural factors can influence the accessibility and feasibility of participation in energy-transition. Interviewees emphasise that people experience different barriers to participate in workshop formats, informational events or online meetings which relate to financial and time resources, and the role of technology.

Time was acknowledged as a significant barrier, particularly for individuals in precarious living conditions and/ or with care responsibilities:

*“Most events take place in the evening, which makes it more difficult for young families to participate.”* (I.5)

Beyond the number of hours available, interviewees also stress a missing “mental space.” If people are preoccupied with other pressing issues, they are less likely to get involved in the energy transition.

*“I assume it’s mostly people who are already sensitized to the topic—those who have the privilege of having the time to engage with such issues, or the cultural capital to do so.”* (I.10)

Money is likewise perceived as a potential barrier. In the context of balcony solar systems, supportive funding mechanisms and solidarity-based models are often available, yet initiatives report that they may not always be sufficient or may be unknown or inaccessible to those who would benefit most. Complex bureaucratic procedures, missing information or not well targeted outreach are cited as reasons for this. Besides the upfront costs of solar systems, other, more hidden barriers could be attributed to money—for example, the assumption that one would need a car to transport the solar panels, even in projects where the panels themselves would be provided free of charge.

Property ownership, often a precondition in renewable energy participation, is less critical in the case of balcony solar systems. However, resistance from homeowners’ association or landlords is mentioned as a significant barrier, which affects people in precarious rental situations more than property owners.

Technology emerges in interviews both as a perceived barrier and as a locus of gendered socialisation. In all cases, interviewees emphasise that no prior technical knowledge or craft skills were necessary for participation in trainings, workshops, or events. However, they acknowledge that the widespread assumption of such a requirement poses a barrier—particularly for FLINTA*—and may lead to forms of *“self-exclusion”* (I.12).

*“But perhaps it's more the fear of a lack of technical knowledge that makes you [women] think: ‘I don't know that, I'm not smart enough, I'm not familiar with it.’”* (I.3)

Some interviewees further suggested that these self-exclusion mechanisms are rooted in gendered socialization processes related to self-esteem—where women tend to underestimate and men tend to overestimate their competencies in technical fields. Others point to socialisation effects that keep girls and women from engaging with energy production or technological topics.

#### 4.1.3 Recognizing and overlooking exclusion

Reflexive accounts also reveal where exclusion is recognised and where it remains under-acknowledged. Many initiatives are aware of their limited reach beyond familiar social circles and of the homogeneity of their usual participants. This ‘bubble effect’ can hinder outreach and is perceived as potentially alienating for people outside the dominant demographic.

Language is seen as crucial in determining who feels addressed and able to participate. Several initiatives acknowledge lacking resources to provide materials and events in multiple languages—especially in multilingual urban areas like Berlin.

*“We did try to host some events in English to include people who don’t speak German. But overall, we haven’t come very far in that regard. Especially in areas like X or Y, we would love to provide information in Arabic or Turkish as well, but we just don’t have the support or resources for that.”* (I.11)

The decision not to address multilingualism was often influenced by the limited capacity of the team and the absence of native speakers. While language barriers were acknowledged, the importance of the participation of people/women of colour was never explicitly mentioned. No initiative reflected on racism or measures to avoid racist behaviour at events.

Additionally, the use of plain or accessible language is rarely addressed explicitly. The reflections are also shaped by implicit assumption about physical ability and normative notions of bodies. Some interviewees note that certain initiatives assumed participants would be physically able to transport and install solar modules.

*“It was (unintendedly) kind of implied in the way we communicated that people would be able to carry and mount the modules themselves.”* (I.12)

Gendered and queer dynamics are likewise only partly recognised. While there is a growing focus on including women and several strategies to do so (see below), many initiatives struggle to create spaces that genuinely include trans, intersex and non-binary individuals, despite well-meaning efforts (see 4.2.2). Interestingly, this issue of lacking inclusion of TIAN* or queer people was seldomly reported by the interviewed initiatives representatives; it became visible more often in subtext or when asked specifically. This asymmetry between explicit commitments to diversity and what remains unnamed or insufficiently reflected is itself an important reflexive finding and sets the stage for the analysis of performative practices in the following section.

### 4.2 Performative dimensions of inclusion

Across all six initiatives, participants described a variety of concrete practices through which inclusion is enacted, ranging from communication and outreach to the design of spaces, formats, and support mechanisms. Interviewees emphasised that these performative measures are central to enabling participation and fostering diversity, yet many also acknowledged the challenges involved. Several noted that they have difficulties, how to structure safe spaces effectively, how to tailor formats for different target groups, or how to provide practical support while managing limited resources. Others reflected on the complexities of integrating technology in ways that build confidence rather than reinforce exclusion. This recognition that performative inclusion is both essential and demanding sets the stage for the following analysis, which examines six interrelated dimensions: communicating inclusion, designing spaces and formats, solidarity and support mechanisms, the role of technology, experiences of discrimination, and the trade-offs inherent in inclusive practice.

#### 4.2.1 Communicating inclusion: language, imagery, and outreach

Communication strategies play a central role in shaping who feels addressed and empowered to participate. Many initiatives critically reflected on their communication and made efforts to reduce barriers, especially for women and other underrepresented groups. A widely shared strategy is the conscious avoidance of technical jargon and highly specialised illustrations. Instead, some initiatives use multicoloured and sometimes playful designs, which are perceived as more inviting than a sober, engineering oriented aesthetics.

The analysis of the websites and social media accounts shows that most initiatives use gender-sensitive language and, at least partly, aim for gender-balanced visual representation by depicting people perceived as women in technical and leading roles. While these practices are not always implemented systematically, they signal a growing awareness of how language and imagery influence accessibility and identification. When gender is addressed explicitly, initiatives tend to focus primarily on women rather than on a broader spectrum of gender identities. Only two initiatives provide dedicated workshops for FLINTA* participants.

The choice of media formats reflects attempts to reach beyond the typical eco-activist or technically oriented audience. Initiatives often rely on simple and low-threshold communication tools, such as posters, flyers, and websites, complemented in some cases by promotions in local newspapers, printed materials in public spaces, email lists, and social media. This multi-channel approach helps address different informational habits and social milieus. School events are used as a format to reach a mixed group of people and to motivate girls to participate in solar projects, especially when workshops are led by women.

#### 4.2.2 Designing spaces and format: venues, safe spaces and target-group specific events

The design and location of events such as workshops, construction sites and camps strongly influence who attends and how safe participants feel. Initiatives emphasised the importance of choosing inclusive and low-threshold venues. Empirically, the type of space clearly shaped participation: for example, a workshop held in a Sparkasse branch attracted “older wealthier men,” whereas a similar event in a local neighbourhood garden drew a more diverse but also more eco-interested crowd. Events hosted in district centres, community initiatives or local MPs’ offices were seen as promising because these actors have diverse local networks and can act as credible intermediaries. Online events were valued as low-threshold entry points, as people “could turn their camera off and just listen,” but they were seen as less effective for deeper engagement and follow-up.

Several initiatives highlighted the potential of more direct, target-group specific outreach—not only to FLINTA* individuals but also to tenants, single parents or people without access to a car. However, such approaches were rarely fully implemented, often due to resource constraints or uncertainty about how to approach these groups effectively. Still, there is reflexive awareness that programmes specifically for tenants tend to reach different, and usually more diverse, people than programmes aimed only at homeowners, and some initiatives have already started tenant-focused formats.

The need to create safe(r) spaces emerges clearly from the reported discriminatory experiences. FLINTA*-specific formats (e.g., FLINTA*-only solar workshops or construction sites) are understood as potentially necessary—not as separation for its own sake but as a form of self-empowerment and protection. However, our data also show that such formats can fall short if they are not grounded in appropriate expertise and awareness. One interviewee who identified as queer described how FLINTA*-labelled spaces can become “traps” when they are de facto safe only for cis women:

“FLINTA* spaces are supposed to be safer for these groups. But if they’re only made up of cis women who aren’t familiar with TIAN* [trans, inter, a-gender, non-binary persons] issues, they may end up being safe for cis women—but not for TIAN* people. That’s often the reality. […] It can feel like a trap—you go there expecting it to be a safe space, and it’s really not.” (I.8)

At the same time, people unfamiliar with queerness may feel overwhelmed and unintentionally hurt queer participants, especially if workshop facilitators are not trained in queer-phobic behaviour or anti-discrimination interventions. One cis-female participant admitted:

“And there was also another group there, what are they called? A FLINTA* group? Exactly. I didn't even know what that was. And I wasn't even prepared for it.” (I.7)

Overall, the absences of clear protocols or response strategies when discrimination occurs, whether through moderation guidelines, feedback systems, or clear accountability structures, represents a major gap in the creation of genuinely inclusive spaces.

Access to participatory formats in the energy transition is shaped by more than time or financial capacity. Language, physical ability, age, race, gender, and social belonging intersect to produce very different experiences of inclusion and exclusion. Recognizing and addressing these contextual factors is key to creating truly inclusive participation.

#### 4.2.3 Solidarity models, practical support, and organisational conditions

To address participation barriers related to finances, bureaucracy, and technical implementation, several initiatives adopted solidarity-based models and practical support strategies. These include contribution schemes where those with more financial resources pay more, enabling others to pay less, as well as collective efforts where volunteers support installation work or accompany others through the process.

Many initiatives offer support with funding applications, guiding participants through complex bureaucratic procedures for balcony solar panel support funding. One initiative began collaborating with a housing cooperative and considered working with a social welfare organisation to simplify access to support structures for private energy production.

Hands-on formats, such as building solar systems together, were perceived as particularly effective, as they enabled learning through practice and fostered a sense of shared ownership, especially for those who lacked technical confidence. However, as discussed above, the inclusiveness of these formats depended strongly on facilitation and the handling of discriminatory behaviour.

Organisational structures and resource shape the extent to which such practices can be sustained. Initiatives that implemented inclusive practices more consistently tended to have clearer responsibilities, some dedicated resources, formal coordination structures, and a higher level of internal education and awareness around issues of diversity and discrimination. These conditions provide a stronger basis for planning, maintaining, and evaluating inclusion-oriented strategies. Nevertheless, interviewees repeatedly stressed that time and financial constraints pose significant challenges to this work: most initiatives operate with limited budgets and rely heavily on unpaid or voluntary labour. Under such conditions, diversity work often competes with other pressing tasks, leading to practical limitations despite strong normative commitments.

*“We’d definitely be open to it. But it's always a matter of limited capacity. We already have so much to do and can’t manage everything.”* (I.3)

Gendered division of labour also appears as a structural constraint. Technology oriented and decision-making practices were often dominated by cis-men, tasks related to communication, education, and care work were disproportionately often carried out by FLINTA* individuals. Although initiatives usually emphasised the importance of encouraging women and TIAN* persons into technical domains, roles, there was much less reflection on whether men should take on more responsibility in communication, care, or community work or on the need to reflect on or change their own behaviours, which is a major part of exclusion in practice as we will elaborate on in the next two chapters.

#### 4.2.4 Technology in practice: interactions, confidence, and exclusion

Beyond perceived thresholds, technology plays a central role in practice through workshop dynamics and interaction patterns. In all observed events, some male participants asked highly specific and often unnecessarily detailed questions or made extensive comments to highlight their technical knowledge. This behaviour tended to create an atmosphere in which balcony solar appeared as a complex and highly technical subject. Workshop facilitators frequently had to intervene and redirect the discussion to keep it accessible and relevant for all participants. Such dynamics can reinforce the impression that solar technology—and the energy transition more broadly—requires in-depth expertise, thereby discouraging those who already doubt their technical competence.

For FLINTA*, the mere perception that spaces are dominated by ‘male structures’ can act as a powerful deterrent. One interviewee who identified as queer explained:

*“I’m honestly really frustrated with all the male-dominated structures in this field—and I don’t feel like dealing with it anymore. […] As soon as it involves technology, it always seems to be dominated by male structures. And I’m really eager to learn more—but I don’t do it, because I find the people involved so unpleasant.”* (I.8)

Similar experiences were reported by women working in the field. A cis woman involved in telephone consultations recounted:

*“I often have these rather amusing experiences during our telephone consultations. Quite frequently, people say to me: “Yes, could you connect me with one of your male colleagues?”—because they don’t want to talk to me. I always find that especially charming. But I manage. I always try to handle it and steer the conversation in a good direction. And in the end, the call is usually friendly. But there’s just this underlying assumption that, because of my gender, I’m not really competent in this field. That’s unfortunate.”* (I.4)

For queer and trans people, mere presence in the field can still feel exceptional:

“I think it's a significant step forward, even though it's sad that simply existing in this field still feels remarkable or unusual for a trans person.” (I.8)

These accounts underline how sexist and queerphobic structures are not only perceived but enacted in everyday interactions, which lead to experiences of discrimination discussed in the next chapter.

#### 4.2.5 Experiences of discrimination and the limits of inclusion

Despite inclusive intentions, the data reveal multiple discriminatory experiences against FLINTA* in workshops and events. These were ranging from paternalist communication patterns and belittling of technical competence to sexist jokes and queerphobic remarks. In four out of five events, such behaviour of male participants or male facilitators was documented, for example laughing at a woman’s question, assuming an app would mainly interest men, or taking tools out of women’s hands to “show” how to use them. In a workshop with six FLINTA* and fifteen men, two FLINTA* participants reported feeling uncomfortable due to men’s behaviour (including one organiser) but did not feel able to speak to male facilitators; later feedback did not lead to an apology or clear understanding of the problem. Sexist jokes, such the following, were dismissed as humour when challenged:

Instructor *“The minus plug is the female one. Easy to remember, right?”*

(Several male participants laugh.)

FLINTA* participant: *“You do realize there’s a shortage of women in this sector, right? I wonder why that is.”*

Instructor: *“I didn’t come up with the terminology. […] It’s all meant as a joke anyway. Don’t take it seriously.”*

Even FLINTA*-only spaces did not necessarily guarantee safety: a participant who identified as queer described their gender being disacknowledged in such a workshop, which felt particularly hurtful given the event’s framing.

Across cases, initiatives lacked clear protocols for addressing all these incidents and facilitators were unsure how to respond. Overall, representation and targeted outreach alone did not ensure inclusion. Without active strategies against discriminatory behaviour and underlying power dynamics, well-intentioned formats risked reproducing existing exclusions, although several initiatives expressed interest in further training on these issues. Our data thus indicate that while many initiatives are aware of gendered and intersectional barriers, their approaches to addressing them vary widely in depth and consistency. Discriminatory behaviour against women and TIAN* persons is still happening and often already anticipated by them. Representation and targeting diverse groups alone do not guarantee inclusion. Without attention to underlying mechanisms of power and the counteraction of discriminatory behaviour, even well-intentioned formats can reinforce existing exclusions.

#### 4.2.6 Trade-offs of inclusion

Initiatives that actively foreground FLINTA* participation or make diversity work a core concern also report trad-offs with their funding schemes or broader accessibility. Some interviewees voiced concerns that strongly FLINTA*-focused activities might alienate parts of their existing (mostly male) membership and/or would risk further funding.

*“I still wonder, a bit sceptically, how the membership would respond if we said: from now on, we’re mainly doing things for women. Wouldn’t most people feel pushed away? We haven’t tried it yet. We’re not quite there.”* (I.3)

While this is part of a feared backlash by men, tensions can also arise between different target groups around language and framing. Terms such as “FLINTA*” are experienced as powerful tools for visibility and empowerment for queer people but are also seen as potentially confusing or off-putting for people unfamiliar with queer-feminist discourses. Interviewees with more experience in this field emphasised that tensions are not an argument against inclusive terminology but rather call for “translation strategies,” like using the term “women and queer people” alongside short, accessible explanations to bridge the gap between inclusivity and reach. Overall, many initiatives are still experimenting with how to communicate diversity aims in ways that neither dilute their commitments nor unintentionally narrow their audience, highlighting the need for more systematic reflection and shared learning on these trade-offs.

### 4.3 Patterns of reflexive-performative inclusion and transformative depths

Our findings can be synthesised as recurring patterns that link how initiative reflect and talk about gender diversity and justice (reflexive) with how they organise and act in practice (performative). Rather than treating these as fixed types, we describe four dominant logics: “inclusion under climate-first pragmatism,” “inclusion as equal access,” “inclusion as community and care,” and “inclusion as questioning norms and creating safer spaces.” For each pattern, we consider characteristic reflexive orientations, typical practices, and the extent to which these contribute to incremental adjustments or to more far-reaching, architectural changes in the sense of Dolata’s theory of socio-technical transformation (see overview in Table 3).

**Table 3:** Four patterns of reflexive-performative inclusion and transformative depths.

Pattern	Typical sayings (reflexive)	Typical doings (performative)	Transformative depth
Inclusion under climate-first pragmatism	Strong climate and EE expansion focus; inclusion seen as desirable but secondary (“reach more people”), binary concepts	Broad, non-specific outreach; general prosumer offers; limited gender/diversity-specific formats or structural changes	Mainly incremental; risk of reproducing inequalities
Inclusion as equal access	Emphasis on equality and fair participation; focus on “more women” in events and boards; mostly binary framing	Women inclusive language; more diverse imagery; occasional women focused events; limited change in roles or governance	Mostly incremental (addon measures)
Inclusion as community and care	Focus on neighbourhoods, mutual support, shared learning, care for people and environment	Neighbourhood based workshops; joint installations; solidarity fees; help with funding applications; cooperation with local actors	Incremental with some architectural elements
Inclusion as questioning norms/safer spaces	Attempts to move beyond binaries; references to FLINTA* and queer experiences; desire for safe(r) spaces	FLINTA* only workshops/camps; experiments with safer spaces; ad hoc responses to discrimination; few stable procedures	Ambivalent: norm critical but structurally fragile

The first pattern, ‘inclusion under climate-first pragmatism’, prioritises climate neutrality as its primary goal, adopting an ecological-technical approach. Diversity is recognised as important insofar as it helps to achieve this objective. It adopts an open and pragmatic stance toward different forms of involvement. This means that it approaches gender and diversity mainly in line with mainstream norms, adapting when useful for broader participation. Consequently, no specific feminist approaches or justice concepts are applied, which risks the reproduction of inequalities.

In the pattern “inclusion and equal access,” inclusion is primarily articulated as a question of equal access and participation, especially for women. Reflexive practices emphasise that “more women” should be present in workshops, on boards or in membership, and that participation should be “fair” and “open to all,” while gender is mostly framed in binary terms. These reflexive orientations resonate with liberal-feminist ideas of equality, representation and non-discrimination. Correspondingly, doings focus on symbolic and communicative measures. Initiatives use gender-sensitive language in their materials, place more women in images, and avoid overly technical jargon in order to make offerings more approachable. There is little systematic change in who occupies technical or decision-making roles, how responsibilities are distributed, or how internal rules are defined. Overall, these sayings and doings represent incremental inclusion: they expand access within largely unchanged organisational and socio-technical arrangements.

The pattern “inclusion as community and care” understands inclusion primarily through the lens of community, care, and everyday life. Here, initiatives describe the energy transition as something that happens through mutual support, shared learning and care for people and the environment. The reflexive practises highlight themes such as “doing things together,” “helping each other” and “making solar accessible for the whole neighbourhood,” echoing ecofeminist perspectives that link social and ecological justice and foreground relationality. In practice, this orientation is enacted through collective and solidarity-based formats: neighbourhood workshops, solar “parties,” joint installation days, support with funding applications, and solidarity contributions where better-off participants cross-subsidise others. Initiatives seek to cooperate with schools, community centres, housing cooperatives or social welfare actors, thereby embedding balcony solar and citizen energy initiatives in broader local networks. These doings often remain incremental in the sense that formal roles and governance structures change little. Yet where collaborations and solidarity models are stabilised and systematically oriented to tenants or low-income groups, we see elements of architectural change, as participation opportunities and resource flows are reconfigured beyond individual goodwill.

A fourth pattern is characterised by attempts to question gender norms and create safer spaces, particularly for FLINTA* and queer people. In their reflexive practices, some explicitly reference FLINTA* or TIAN*, and articulate a desire for formats that feel safer than conventional, male-dominated energy spaces. These norm-critical discourses draw on postmodern and queer-feminist ideas, even if not always explicitly. Doings in this pattern include FLINTA*-only workshops or solar camps, initial experiments with safer-space guidelines, and the explicit naming of queer participants as desired audiences. However, the empirical material shows that such efforts are ambivalent. FLINTA*-labelled spaces may still be experienced as safe mainly for cis women, while TIAN* participants encounter misrecognition or lack of awareness. Discriminatory behaviour—such as misgendering—is rarely addressed through established protocols; facilitators respond *ad hoc*, if at all, and reported uncertainty about how to intervene. This pattern thus combines relatively far-reaching sayings (norm-critical, intersectional aspirations) with fragile doings that lack institutional anchoring. Transformative depth is accordingly mixed: the potential for architectural change exists but currently remains underrealized.

Across all four patterns, a central observation is that sayings are often more ambitious than doings. Many initiatives articulate strong commitments to inclusion, name barriers in terms of time, money and social belonging, and in some cases invoke intersectional or queer-feminist perspectives. However, the corresponding practices frequently remain at the level of symbolic adjustments, isolated project formats or individual engagement. At all-gender events, FLINTA* often experience various forms of discrimination. While FLINTA*-only events seem to be the only option for cis gender women to attend an event without fearing, anticipating or experiencing discrimination, this is not guaranteed for TIAN* persons. Our empirical observation that, overall, very few TIAN* individuals took part in the workshops may be linked to the fact that the way they are addressed and the services on offer are not yet sufficiently tailored to their needs, and that the relevant target groups may already anticipate discrimination. From the perspective of transformative depth, incremental change is widespread and important: it improves access, visibility and recognition within existing structures. Architectural transformation, in contrast, requires that reflexive re-articulations of inclusion and justice are coupled with durable changes in roles, rules, resources and relationships, for instance, by redistributing technical and decision-making responsibilities, institutionalising safer-space and anti-discrimination practices, and stabilising collaborations with actors who represent marginalised groups. The patterns described here suggest that citizen energy initiatives currently stand at different points along this continuum, with most still operating in incremental modes and a few beginning to experiment with more structurally transformative ways of inclusion.

## 5 Discussion

While all initiatives are doing pioneering work and express a strong willingness to expand their diversity measures, these ambitions remain limited in practice. Most initiatives tend to overestimate their level of diversity, which is a common issue in terms of organisation diversity (Ahmed, 2012). We identified three main reasons for this, financial constraints, the role of technology and patriarchy, and forms of masculinities common in CE initiatives, which are discussed in the following subchapters.

### 5.1 Practical and financial constraints

Similar to findings by Ely and Thomas (2001), our results suggest that commitment alone is insufficient when structural and organisational capacities are lacking. As one interviewee noted, *“there are many barriers that we are not even aware of, and addressing them requires enormous and targeted communication efforts”* (I.12). Financial precarity further reinforces these limitations, as several interviewees highlighted the strain of limited time and overstretched teams. Funding structures may even create tensions between mission and inclusion efforts, as illustrated by one initiative:

“It would be easier to receive funding if we opened the Solar Camp to all genders, but maintaining our focus [on FLINTA*] is important to us, so we try to overcome the resistance.” (I.2)

This points to the importance of organisational structures and resource availability for shaping the strategic capacity to implement diversity measures. Initiatives with clearer responsibilities and dedicated resources are better positioned to translate intentions into concrete strategies, whereas informal and volunteer-based structures create an ambivalent environment: they enable openness, but they also limit commitment, continuity and accessibility.

Taken together, these findings indicate that the gap between diversity ambitions and actual implementation is shaped less by a lack of willingness and more by shortcomings in money, time and priority setting. All initiatives rely on unpaid or only sporadically compensated work, and many interviewees emphasized that diversity and inclusion are treated as additional tasks driven by individual motivation rather than being embedded as an inherent part of the organisational structure. Moreover, even where diversity is acknowledged as highly important from a reflexive perspective, implementation is often constrained by organisational architectures, limited resources, and broader structural conditions under which initiatives operate. Thus, initiatives would need increased and more secure state or non-state funding in order to enhance their diversity.

### 5.2 The role of technology or rather of patriarchy?

In both academic literature and public discourse, the energy transition is largely presented as a technical issue (Łapniewska, 2019). This technocentric framing is often cited as a reason for the underrepresentation of women in the energy sector, based on the assumption that women are less interested in technical topics or have lower knowledge in this field because of socialization and education (i.e., Lazoroska et al., 2021; Mechlenborg and Gram-Hanssen, 2022). An educational background in, or at least an interest in, STEM subjects (science, technology, engineering, and mathematics) is frequently assumed as a prerequisite for taking part in the energy-transition.

In contrast, our findings suggest that engaging in CE initiatives typically does not require prior technological expertise. Nevertheless, the persistent perception that renewable energy and solar technologies are inherently technical and thereby mostly male-dominated can be intimidating and discourage the participation of FLINTA*, even when detailed technical knowledge (e.g., how solar panels work) is not actually required. Most of the initiatives are aware of that and try to counteract the impression of the technical character due to using illustrations in their material that are explicitly not technical and are partly successful with this.

Additionally, our findings indicate that a major driver of exclusion – largely unrelated to technology itself – is discriminatory or uncomfortable behaviour, particularly by men, as well as the anticipation or fear of encountering such behaviour in technical, and thus often male-dominated, spaces. This aligns with studies pointing to the psychological burden of patriarchal structures, showing that women’s short retention in STEM is often due to “gendered practices and conventions” shaped by male-dominated power relations (Solga and Pfahl, 2009). From this perspective, exclusion is shaped less by technological barriers than by patriarchal social relations embedded within these contexts. In summary, our results suggests that the idea of a lack of education or interest in STEM as a barrier for FLINTA* to participate in CE is more socially constructed than empirically substantiated. Thus, we contend that the issue lies with patriarchy, not technology.

Besides overcoming patriarchal and sexist structures in organizations, we call for a re-conceptualization of technology as a caring practice for human and nature. If technology is reframed as an invitation for collective action, its underlying mechanisms and related knowledge can be de-dramatized and demystified. In doing so, this approach could both broaden participation and shift the understanding of technology away from being an end in itself toward being a means to socially defined goals as, i.e., climate neutrality or renewable energy coverage.

### 5.3 Between eco-modern and ecological masculinity

As we found that the behaviour of men is one of the major drivers of exclusion, the issue of present masculinities in CE arises. While the forms of masculinity observed in the initiatives clearly differ from *petromasculinity* (Daggett, 2018) in terms of their fossil and authoritarian orientation, they cannot be unambiguously assigned to either of the two alternative forms proposed by Hultman and Pulé (2018), namely *eco-modern* or *ecological masculinities*. Following Hultman’s (2021) argument, one might expect men engaged in citizen-led renewable energy initiatives to more closely enact *ecological masculinities*, characterized by “*the localisation of communities, the prioritisation of social over economic relationships, the use of small-scale technology, the operation of renewable energy conversion, the decentralisation of power structures, and living in harmony with nature as a central part of everyday life”* (Hultman, 2021:16). This would include men educating themselves about patriarchy and discrimination, creating opportunities, relinquishing control and actively addressing discriminatory patterns within the group, ranging from dominant speech habits to dismissive attitudes.

While many of these attributes are indeed reflected in the initiatives we studied, our findings also reveal the persistence of gendered practices that resonate with core elements of *petro-* and *eco-modern masculinity*, particularly male autonomy, control, and guidance (Daggett, 2018; Hultman and Pulé, 2018). Importantly, these elements do not manifest through fossil-fuelled dominance, overtly authoritarian politics, or open sexist assumptions, but rather through subtler, morally legitimized and socially embedded practices within renewable energy contexts. In most initiatives, communication, educational work, and care-related tasks are predominantly carried out by women (see chapter 4.2.3) whereas men more frequently occupy formal or informal leadership positions, for instance as workshop facilitators or technical experts. In addition, various forms of discriminatory behaviour by men toward FLINTA* were observed across many settings. Although men involved in the initiatives are highly committed to ecological issues and the renewable energy transition, these practices stand in marked contrast to the relational, care-oriented form of ecological masculinity proposed by Hultman and Pulé (2018). They reflexively may appreciate and value diversity in theory but are somehow unable to perform accordingly.

Configurations of masculinities we found empirically seem to be stuck between eco-modern and ecological masculinity, and still reproduce autonomy, control, authority, and leadership within renewable and ecological oriented energy initiatives. To capture this configuration, we propose the concept of ecological-hegemonic masculinity. Unlike Daggett’s petromasculinity, which is centred on fossil-fuelled domination and resistance to decarbonization, ecological-hegemonic masculinity operates through ecological moral authority (“doing the right thing” by engaging in renewable energy), technical autonomy (“doing it yourself” for example by independently installing balcony solar systems), situational control, and guiding or instructive roles within group settings. Taken together, these practices reconfigure hegemonic masculinity (Connell and Messerschmidt, 2005), often reproducing asymmetries of power and implicit superiority over FLINTA* rather than dismantling them, within ostensibly progressive and sustainable spaces. In this sense, ecological-hegemonic masculinity differs from the normative ideal of *ecological masculinity* proposed by Hultman (2021), raising the question of whether such a fully relational and non-hierarchical form of masculinity can be realised in practice under current socio-structural conditions.

At the same time, ecological-hegemonic masculinity should not be understood as inherently harmful or intentionally exclusionary. Rather, it highlights how even well-intentioned, environmentally progressive people involved in CE initiatives can unintentionally reproduce hierarchies and exclusions, particularly when leadership, expertise, and decision-making remain unevenly distributed along gendered lines. From this perspective, the participation of FLINTA* is crucial for challenging these diverse forms of masculinities and thereby socio-structural conditions. We therefore emphasise the importance of FLINTA*-only spaces and other protected formats that provide safer spaces for exchange, collective reflection, peer-empowerment and capacity strengthening. Such spaces can be understood not as contradicting diversity goals, but as a necessary precondition for achieving more inclusive and genuinely diverse participatory settings in the long term.

## 6 Conclusion

This study demonstrates that high normative aspirations regarding diversity stand in contrast to persistent practical constraints. Beyond organisational factors, like limited finances and capacities, our analysis identifies two central socio-cultural dynamics that continue to hinder diversity. First, technology in combination with patriarchy remains a largely “closed shop” for FLINTA*. While many initiatives attempt to counter this by reframing energy transitions and PV as caring and socially embedded practices, socially ingrained underestimations of one’s own technical competence, coupled with the anticipation or experience of male-dominated technical spaces and discriminatory behaviour, continue to pose significant barriers to participation for FLINTA*. Second, our findings reveal that forms of masculinity within the initiatives—while often explicitly committed to inclusivity—frequently reproduce exclusionary practices in everyday interactions.

Theoretically, this study contributes a novel perspective distinguishing between reflexive and performative approaches to diversity and inclusion in CE initiatives and connects these to different forms of transformative depths. Empirically, our findings challenge assumptions that citizen-led renewable energy initiatives necessarily foster the “*ecological masculinities*” proposed by Hultman and Pulé (2018). Instead, we introduce the concept of ecological-hegemonic masculinity to describe a configuration in which men (and masculine-coded identities), despite valuing renewable energy, social over economic benefits, and care-oriented narratives, continue to align with hegemonic masculine norms of autonomy, control, and guidance. Ecological-hegemonic masculinity thus differs from ecological masculinity by showing how hegemonic gender relations are reconfigured but not dissolved within ostensibly progressive and sustainable contexts.

Future research should further investigate how different forms of masculinity emerge and interact within just energy transitions and critically examine whether and under what conditions masculinities can actively support diversity and inclusion rather than undermine them. Comparative studies across energy sectors, organisational forms, and cultural contexts could provide valuable insights into the variability and transformability of gendered power relations in sustainability transitions. Furthermore, the perspectives of individuals facing intersectional discrimination, including TIAN*, FLINTA* of colour and disabled FLINTA*, should be given more attention. These groups are underrepresented in our study.

From a practical perspective, our findings highlight the importance of creating protected and safer spaces for FLINTA* and other marginalized groups within energy initiatives. Such spaces can provide opportunities to engage with technical and energy-related topics without the immediate risk of exclusionary or discriminatory dynamics, thereby supporting confidence-building, skill development, and collective empowerment. At the same time, our results highlight the need for sustained financial and institutional support of DIY-PV-initiatives, as only adequate resources and capacities can move diversity and inclusion from individual commitment to a structurally embedded practice.

## Data Availability

Due to the sensitive nature of the qualitative data, the raw data are not publicly available. Anonymized excerpts may be provided upon reasonable request.

## Footnotes

1 Since 2025, titled BMWE.

## References

[ref1] Ahlborg H. Michael K. Unsworth S. J. Hategekimana S. Osunmuyiwa O. Åberg A. . (2024). Thirty-five years of research on energy and power: a landscape analysis. Renew. Sust. Energ. Rev. 199:114542. doi: 10.1016/j.rser.2024.114542

[ref2] Ahmed S. (2012). On Being Included: Racism and Diversity in Institutional Life. Durham: Duke University Press.

[ref3] Ahmed S. (2017). Living a Feminist Life. Durham: Duke University Press.

[ref4] Barry J. Hume T. Ellis G. Curry R. (2015). “Low carbon transitions and post-fossil fuel energy transformations as political struggles: analysing and overcoming ‘carbon lock-in’,” in Energy & Environmental Transformations in a Globalizing World: An Interdisciplinary Dialogue (Athens: Nomiki Bibliothiki), 3–23.

[ref5] Bell S. E. Daggett C. Labuski C. (2020). Toward feminist energy systems: why adding women and solar panels is not enough. Energy Res. Soc. Sci. 68:101557. doi: 10.1016/j.erss.2020.101557

[ref6] Bidwell D. Sovacool B. K. (2023). Uneasy tensions in energy justice and systems transformation. Nat. Energy 8, 317–320. doi: 10.1038/s41560-023-01217-8

[ref7] Blome-Drees J. Degens P. Flieger B. Lapschieß L. Lautermann C. Moldenhauer J. . (2024). Kooperatives Wirtschaften in der Zivilgesellschaft: gemeinwohlorientiert, tragfähig und transformativ. Frankfurt New York: Campus Verlag.

[ref8] Bögel P. M. Trenks H. Upham P. Sauter H. Albiez M. Stelzer V. . (2023). Diversifying power in action: a socio-psychological approach to inclusive energy transition experiments. Energy Res. Soc. Sci. 100:103070. doi: 10.1016/j.erss.2023.103070

[ref9] Büchs M. Cass N. Mullen C. Lucas K. Ivanova D. (2023). Emissions savings from equitable energy demand reduction. Nat. Energy 8, 758–769. doi: 10.1038/s41560-023-01283-y

[ref10] Cannon C. E. B. Chu E. K. (2021). Gender, sexuality, and feminist critiques in energy research: a review and call for transversal thinking. Energy Res. Soc. Sci. 75:102005. doi: 10.1016/j.erss.2021.102005

[ref11] Connell R. W. Messerschmidt J. W. (2005). Hegemonic masculinity: rethinking the concept. Gend. Soc. 19, 829–859. doi: 10.1177/0891243205278639

[ref12] Crenshaw K. (1989). Demarginalizing the intersection of race and sex: a black feminist critique of antidiscrimination doctrine, feminist theory and antiracist politics. Univ. Chic. Legal Forum 1989, 139–167.

[ref13] Crenshaw K. (1991). Mapping the margins: intersectionality, identity politics, and violence against women of color. Stan. Law Rev. 43:1241. doi: 10.2307/1229039

[ref14] Daggett C. (2018). Petro-masculinity: fossil fuels and authoritarian desire. Millennium 47, 25–44. doi: 10.1177/0305829818775817

[ref15] Dolata U. (2011). Soziotechnischer Wandel als graduelle Transformation. Berl. J. Soziol. 21, 265–294. doi: 10.1007/s11609-011-0153-0

[ref16] Dudka A. (2024). “Women in energy communities: an intersectional analysis of their participation,” in Women and the Energy Sector: Gender Inequality and Sustainability in Production and Consumption, eds. Rocha Lawton N. Forson C. (Cham: Springer International Publishing), 243–262.

[ref17] Ely R. J. Thomas D. A. (2001). Cultural diversity at work: the effects of diversity perspectives on work group processes and outcomes. Adm. Sci. Q. 46, 229–273. doi: 10.2307/2667087

[ref18] Fathallah J. Pyakurel P. (2020). Addressing gender in energy studies. Energy Res. Soc. Sci. 65:101461. doi: 10.1016/j.erss.2020.101461

[ref19] Feenstra M. Özerol G. (2021). Energy justice as a search light for gender-energy nexus: towards a conceptual framework. Renew. Sust. Energ. Rev. 138:110668. doi: 10.1016/j.rser.2020.110668

[ref20] Fraune C. (2015). Gender matters: women, renewable energy, and citizen participation in Germany. Energy Res. Soc. Sci. 7, 55–65. doi: 10.1016/j.erss.2015.02.005

[ref21] Fraune C. (2018). “Bürgerbeteiligung in der Energiewende – auch für Bürgerinnen?” in Handbuch Energiewende und Partizipation, eds. Holstenkamp L. Radtke J. (Wiesbaden: Springer Fachmedien Wiesbaden), 759–767.

[ref22] Ganz K. Hausotter J. (2020). Intersektionale Sozialforschung. Bielefeld: Transcript Verlag.

[ref23] George K. Kludas S. Prehm N. Wolff C. (2018). Gender und Nachhaltigkeit zusammendenken: Warum wir eine feministische Perspektive brauchen. Ökol. Wirtsch. - Fachz. 3, 14–15. doi: 10.14512/OEW330314

[ref24] Giddens A. (1984). The Constitution of Society: Outline of the Theory of Structuration. Cambridge [Cambridgeshire]: Polity Press.

[ref25] Goedkoop F. Jans L. Perlaviciute G. Hamann K. R. S. (2025). Inclusive community energy initiatives? Understanding involvement of different socio-demographic groups. Energy Res. Soc. Sci. 125:104104. doi: 10.1016/j.erss.2025.104104

[ref26] Groneweg K. Habersbrunner K. (2024). Energiewende = Gerechtigkeitswende – ein Blick über den Quotenrand hin zur feministischen Vision FES Impuls. Friedrich-Ebert-Stiftung.

[ref27] Haraway D. (1996). “Situiertes Wissen. Die Wissenschaftsfrage Im Feminismus Und Das Privileg Einer Partialen Perspektive,” in Vermittelte Weiblichkeit: feministische Wissenschafts- und Gesellschaftstheorie, ed. Scheich E. (Hamburg: Hamburger Edition), 217–248.

[ref28] Hoicka C. (2023). How do we practice equity, diversity and inclusion in sustainable energy research? Advice for modern researchers. Energy Res. Soc. Sci. 97, 1–8. doi: 10.1016/j.erss.2023.102964

[ref29] Hoicka C. E. Savic K. Campney A. (2021). Reconciliation through renewable energy? A survey of indigenous communities, involvement, and peoples in Canada. Energy Res. Soc. Sci. 74:101897. doi: 10.1016/j.erss.2020.101897

[ref30] Hooks B. (2012). Where We Stand. 1st Edn. Milton Park: Routledge.

[ref31] Hultman M. (2021). Ökologische Männlichkeiten: Auf Dem Weg Zu Einer Neuen Normalität? ZDfm - Zeitschrift Für Diversitätsforschung Und -Management 6, 8–22. doi: 10.3224/zdfm.v6i1.02

[ref32] Hultman Martin Pulé Paul M. (2018). Ecological Masculinities: Theoretical Foundations and Practical Guidance. 1st edn. Abingdon, Oxon: New York, NY, Routledge, 2018. | Series: Routledge studies in gender and environments: Routledge.

[ref33] Jenkins K. McCauley D. Heffron R. Stephan H. Rehner R. (2016). Energy justice: a conceptual review. Energy Res. Soc. Sci. 11, 174–182. doi: 10.1016/j.erss.2015.10.004

[ref34] Kanning H. Mölders T. Hofmeister S. (2016). Gendered Energy – Analytische Perspektiven Und Potenziale Der Geschlechterforschung Für Eine Sozial-Ökologische Gestaltung Der Energiewende Im Raum. Raumforsch. Raumordn. 74, 213–227. doi: 10.1007/s13147-016-0392-9

[ref35] Karakislak I. Sadat-Razavi P. Schweizer-Ries P. (2023). A cooperative of their own: gender implications on renewable energy cooperatives in Germany. Energy Res. Soc. Sci. 96:102947. doi: 10.1016/j.erss.2023.102947

[ref6006] Kelle U. Kluge S. (2010). Vom Einzelfall zum Typus: Fallvergleich und Fallkontrastierung in der qualitativen Sozialforschung. Wiesbaden: Springer.

[ref36] Krell G. Sieben B. (2011). “Diversity Management: Chancengleichheit Für Alle Und Auch Als Wettbewerbsvorteil,” in Chancengleichheit Durch Personalpolitik: Gleichstellung von Frauen Und Männern in Unternehmen Und Verwaltungen, 155–174.

[ref37] Kuckartz U. (2018). Qualitative Inhaltsanalyse. Mathoden, Praxis, Computerunterstützung, vol. 4 Weinheim and Basel: Beitz Juventa.

[ref38] Laborgne Pia Radtke Jörg. (2023). ‘Akzeptanz und Partizipation in der Energiewende’. Pp. 1–15 in Handbuch Umweltsoziologie, edited by Sonnberger M. Bleicher A. Groß M.. Wiesbaden: Springer Fachmedien.

[ref39] Łapniewska Z. (2019). Energy, equality and sustainability? European electricity cooperatives from a gender perspective. Energy Res. Soc. Sci. 57:101247. doi: 10.1016/j.erss.2019.101247

[ref40] Lazoroska D. Palm J. Bergek A. (2021). Perceptions of participation and the role of gender for the engagement in solar energy communities in Sweden. Energy Sustain. Soc. 11:35. doi: 10.1186/s13705-021-00312-6, 34660168 PMC8506071

[ref41] Lenz I. (2019). “Feminismus: Denkweisen, Differenzen, Debatten,” in Handbuch Interdisziplinäre Geschlechterforschung, eds. Kortendiek B. Riegraf B. Sabisch K., vol. 65, Geschlecht und Gesellschaft (Wiesbaden: Springer Fachmedien Wiesbaden), 231–241.

[ref42] Lorber J. (2010). Gender Inequality: Feminist Theories and Politics. 4th Edn New York: Oxford Univ. Press.

[ref43] Luh Victoria Löw Beer David (2021). Auszubildende im Lausitzer Strukturwandel. Gute Arbeit, sichere Lebensplanung, regionale Verbundenheit.

[ref44] Martinez-Reyes A. Wolters S. Luijk O. Okur Ö. Hoppe T. (2025). Who is vulnerable in regional energy transitions? An intersectional energy justice analysis of the Rotterdam-the Hague region. Energy Res. Soc. Sci. 119:103859. doi: 10.1016/j.erss.2024.103859

[ref45] Mechlenborg M. Gram-Hanssen K. (2022). Masculine roles and practices in homes with photovoltaic systems. Build. Cities 3, 638–652. doi: 10.5334/bc.211

[ref46] Mies M. Shiva V. (2016). Ökofeminismus: Die Befreiung Der Frauen, Der Natur Und Unterdrückter Völker, vol. 1 Neu Ulm: AG SPAK.

[ref47] Mishra R. K. (2013). Postcolonial feminism: looking into within-beyond-to difference. Int. J. English Lit. 4, 129–134. doi: 10.5897/IJEL12.165

[ref48] PwC. (2025). Frauen in Der Energiewirtschaft Gleichberechtigung, Führungsstrukturen, Zukunftsperspektiven.

[ref49] Radtke J. Ohlhorst D. (2021). Community energy in Germany – bowling alone in elite clubs? Util. Policy 72:101269. doi: 10.1016/j.jup.2021.101269

[ref50] Scharnigg R. Martin A. (2024). Gendered sociotechnical imaginaries: understanding the role of masculinities in Portugal’s pursuit of multi-scalar solar energy transitions. Energy Res. Soc. Sci. 108:103372. doi: 10.1016/j.erss.2023.103372

[ref51] Schultz I. Schramm E. Hummel D. (2015). “Gender als Integrationsdimension in der transdisziplinären SÖF (sozial-ökologischen Forschung),” in Nachhaltigkeit anders denken: Veränderungspotenziale durch Geschlechterperspektiven, eds. Katz C. Heilmann S. Thiem A. Moths K. Koch L. M. Hofmeister S. (Wiesbaden: Springer Fachmedien), 217–230.

[ref52] Schwickert Samantha Strikker Paul Lautermann Christian Jaeger-Erben Melanie Preis Astrid Hirschl Bernd. (2025). Gelingensbedingungen für eine gerechte und inklusive ‘Energiewende von unten’: intersektionale und feministische Perspektiven auf Bürger*innen -Energie. Schriftenreihe des IÖW. Berlin: Institut für ökologische Wirtschaftsforschung GmbH, gemeinnützig.

[ref53] Sharma S. E. Das R. R. Janzwood A. Joshi N. MacArthur J. L. Savvidou G. (2025). Equity, diversity and inclusion promises, exclusive practices? How to move towards effective and just energy transitions. Energy Res. Soc. Sci. 120, 1–8. doi: 10.1016/j.erss.2025.103935

[ref54] Shejale S. Zhan M. X. Sahakian M. Aleksieva R. Biresselioglu M. E. Bogdanova V. . (2025). Participation as a pathway to procedural justice: a review of energy initiatives across eight European countries. Energy Res. Soc. Sci. 122:103982. doi: 10.1016/j.erss.2025.103982

[ref55] Siciliano G. Wallbott L. Urban F. Nguyen Dang A. Lederer M. (2021). Low-carbon energy, sustainable development, and justice: towards a just energy transition for the society and the environment. Sustain. Dev. 29, 1049–1061. doi: 10.1002/sd.2193

[ref56] Solga H. Pfahl L. (2009). Doing Gender Im Technisch-Naturwissenschaftlichen Bereich. Discussion Paper SP I 2009-502. Berlin: Wissenschaftszentrum Berlin für Sozialforschung.

[ref57] Søraa R. A. Anfinsen M. Foulds C. Korsnes M. Lagesen V. Robison R. . (2020). Diversifying diversity: inclusive engagement, intersectionality, and gender identity in a European social sciences and humanities energy research project. Energy Res. Soc. Sci. 62:101380. doi: 10.1016/j.erss.2019.101380

[ref58] Sovacool B. K. Bell S. E. Daggett C. Labuski C. Lennon M. Naylor L. . (2023). Pluralizing energy justice: incorporating feminist, anti-racist, indigenous, and postcolonial perspectives. Energy Res. Soc. Sci. 97:102996. doi: 10.1016/j.erss.2023.102996

[ref59] Tsagkari M. (2022). The need for gender-based approach in the assessment of local energy projects. Energy Sustain. Dev. 68, 40–49. doi: 10.1016/j.esd.2022.03.001

[ref60] Van Uffelen N. (2022). Revisiting recognition in energy justice. Energy Res. Soc. Sci. 92:102764. doi: 10.1016/j.erss.2022.102764

[ref61] Von Kemfert C. Egerer O. (2017). Frauen sind in Top-Positionen der größten Energie- und Verkehrsunternehmen in Deutschland deutlich unterrepräsentiert. DIW Wochenber. 47, 1070–1080.

[ref62] Walk P. (2024). From parity to degrowth: unpacking narratives of a gender-just transition. Energy Res. Soc. Sci. 112:103513. doi: 10.1016/j.erss.2024.103513

[ref63] WECF (2020). Frauen. Energie. Wende! Warum Wir Eine Geschlechtergerechte Energiewende Brauchen. München: Women Engage for a Common Future e.V. (WECF).

[ref64] Winker G. Degele N. (2009). Intersektionalität: Zur Analyse Sozialer Ungleichheiten. transcript. Bielefeld: Verlang.

[ref65] Witzel A. Reiter H. (2012). The Problem-Centred Interview: Principles and Practice. 1 Oliver’s Yard, 55 City Road, London EC1Y 1SP United Kingdom: SAGE Publications Ltd.

[ref66] Wolfram M. Kienesberger M. (2023). Gender in sustainability transition studies: concepts, blind spots and future orientations. Environ. Innov. Soc. Transit. 46:100686. doi: 10.1016/j.eist.2022.100686

[ref67] Yildiz Ö. (2014). Financing renewable energy infrastructures via financial citizen participation – the case of Germany. Renew. Energy 68, 677–685. doi: 10.1016/j.renene.2014.02.038

